# Acoustics of rubbing feathers: the velvet of owl feathers reduces frictional noise

**DOI:** 10.1242/jeb.246234

**Published:** 2025-01-27

**Authors:** Lori G. Liu, Christopher J. Clark

**Affiliations:** Department of Evolution, Ecology, and Organismal Biology, University of California, Riverside, Riverside, CA 92521, USA

**Keywords:** Aeroacoustics, Quiet flight, Pennulums, Locomotion-induced sound, Sonation, Strigiformes

## Abstract

One feather structure associated with an owl's ability to fly quietly is the soft dorsal surface on their flight feathers: the velvet. This velvet is a mat of elongated filamentous pennulums that extend up from feather barbules. The aerodynamic noise hypothesis posits this velvet reduces aerodynamic noise caused by the formation of turbulence, while the structural noise hypothesis posits the velvet acts as a dry lubricant, reducing frictional noise produced by feathers sliding past one another. We investigated the structural noise hypothesis by quantifying the length of the velvet on 24 locations across the wing of the barred owl (*Strix varia*) and then qualitatively assessing the presence of velvet in 24 bird species. We found that velvet has evolved at least 4 times independently (convergently) in owls, nightbirds, hawks and falcons. Then, we rubbed 96 pairs of feathers together from 17 bird species (including the four clades that have independently evolved velvet) under three experimental treatments: control, hairspray applied (to impair the velvet) and hairspray removed. The sound of feathers rubbing against each other was broadband, similar to the sound of rubbing sandpaper or Velcro. Species with velvet produced rubbing sounds that were 20.9 dB quieter than species without velvet, and velvet-coated feathers became 7.4 dB louder when manipulated with hairspray, while feathers lacking velvet only increased in loudness by 1.7 dB, relative to the control treatments. These results all support the hypothesis that the velvet primarily functions to ameliorate the sounds of feathers rubbing against other feathers.

## Teaser

**Summary:** Experimentally rubbing isolated feathers together shows that the velvet on owl feathers substantially diminishes the sound of feathers rubbing against neighboring feathers.

## INTRODUCTION

Quiet flight is a feature of owl hunting ecology. Flying quietly may allow owls to go undetected by prey (stealth) or enable them to better hear prey (Clark et al., 2020a). One of the derived wing features thought to reduce noise produced by flight is the velvet, which is a coating of elongated feather filaments on the upper surface of flight (remiges and rectrices) and covert feathers that gives them a softer feel than regular feathers (Bachmann et al., 2007, 2012). These elongated filaments were additionally observed by Matloff et al. (2020) on the underlapping (lower) surface of certain barn owl wing feathers (see their fig. 4D). In many owls, this velvet resembles napping in fabric. Close examination reveals that the velvet is composed of individual small (micrometer to millimeter scale), elongated and flexible filaments, termed pennulums, which vary in density and length on flight feathers (Lucas and Stettenheim, 1972; Carlisle, 1925; Chandler, 1916).

Matloff et al. (2020) documented atonal sound caused by pigeon (*Columba livia*) feathers rubbing together. In pigeon feathers, these sounds were due to small asperities on overlapping feathers. These sounds are largely absent when barn owl feathers rub together in a similar configuration. Older work called these asperities ‘friction barbules’ (Lucas and Stettenheim, 1972; Graham, 1934), but Matloff et al. (2020) demonstrated that the mechanism preventing slipping is actually interlocking rather than friction, i.e. the asperities function to hold overlapping feathers together but act less like sliding sandpaper (friction) and more like sliding Velcro (interlocking). Matloff et al. (2020) showed that owl feathers lack these asperities and instead have velvet, suggesting that the velvet may have evolved from these small asperities.

There are two hypotheses for how this velvet reduces the acoustic signature of flight. One hypothesis is that the velvet modifies the turbulent boundary layer on the wing in a way that reduces the amount of aerodynamic noise produced (Lilley, 1998; Klän et al., 2012; Kroeger et al., 1972; Jaworski and Peake, 2013). For instance, the velvet may have a spatial effect, elevating the location of turbulence formation away from the surface of the wing (Jaworski and Peake, 2020) and/or it might have a spectral effect, changing the spectrum of vorticity as it forms on the surface of the wing, either of which might affect the sound produced. We call this hypothesis the aerodynamic noise hypothesis (ANH). Alternatively, the velvet might reduce frictional sounds such as those produced by feathers rubbing against other objects during flight, especially other wing feathers (Lucas and Stettenheim, 1972; Clark et al., 2020b; Matloff et al., 2020). This structural noise hypothesis (SNH) proposes that the velvet reduces the noise produced by feathers sliding past one another, by behaving as a dry lubricant. In support of this hypothesis, manipulation of the velvet on inner wing feathers of live barn owls (*Tyto alba*) caused more sound to be produced during upstroke (LePiane and Clark, 2020). Moreover, Clark et al. (2020b) qualitatively scored the presence of velvet on a 3-level ordinal scale (absent, short, long) on spread wings of 50 bird species at a museum (but, because these were museum specimens, they did not conduct more invasive measurements to quantify the length of the pennulum) and found that for all species that had the velvet, it was longest in regions where feathers overlap, in support of the SNH.

Here, we sought to expand on this work. The SNH and the ANH make contrasting predictions (Table 1), four of which we tested. Prediction 1: if the velvet is to reduce aerodynamic noise, we predicted it would be located in feather regions exposed to air during flight (mainly exposed parts of the primaries and secondaries, wing tips of individual feathers, and outer feather vanes). By contrast, if the velvet functions to reduce frictional noise, it should be longest in areas where feathers rub together (portions of the primaries and secondaries that overlap with adjacent feathers, basal regions of individual feathers, and inner feather vanes).

**Table 1.** Predictions made by the aerodynamic noise and structural noise hypotheses

Source of sound	Scenario		Structural noise hypothesis	Aerodynamic noise hypothesis
Distribution of velvet across the wing	Velvet present	1	Elongated velvet in feather regions that rub	Elongated velvet in exposed feather regions that experience turbulence
Sound properties	N/A	2	Broadband; similar sound levels across many frequencies	Broadband, but predominantly low frequency; sound declines at higher frequencies
Experimentally rubbing feathers	Velvet absent	3	Broadband sound; and sound matches flight sounds	Rubbing sounds may not match spectral properties of flight sounds
	Velvet present	4	Quieter than when velvet absent; broadband and matches flight sounds of owl	Rubbing sounds may not match spectral properties of flight sounds
Bird flight	Velvet absent	5	Gliding is quiet relative to flapping flight	Gliding and flapping produce similar sound levels (assumes similar turbulence)
	Velvet present	6	Flapping and gliding produce similar sound; quieter than birds lacking velvet	Gliding and flapping produce similar sound levels; quieter than birds without velvet

Prediction 2: the two hypothesized source mechanisms produce sound with different spectral qualities; therefore, we predicted that while both structural sound and aerodynamic sound present as broadband (i.e. not tonal, but with sound present across many frequencies), aerodynamic sound would tend to peak at a low frequency, with sound power levels tapering off as frequency increases (Blake, 2017), similar to ‘brown’ or ‘pink’ noise. By contrast, in frictional noise, the sound power levels may be evenly distributed across a broad range of frequencies, i.e. resembling white noise. To test this prediction, we recorded the sound of sandpaper and Velcro (structural noise, representing friction and interlocking) and a feather that was whipped past a microphone to generate aerodynamic noise. We compared these recordings with our feather rubbing sounds.

Prediction 3: while both the SNH and ANH predict an overall quieting effect of the velvet on flight sounds, the SNH specifically predicts that flight sounds are produced by feathers rubbing together; thus, experimentally rubbing isolated feathers together should produce broadband sound that resembles flight sounds of actual birds. Prediction 4: as the velvet is hypothesized to reduce flight sounds, we predicted that experimental manipulation of the velvet would affect rubbing sounds: feathers with velvet intact will produce quieter rubbing sounds than feathers without velvet. We sought to test prediction 4 using a simple experimental paradigm: examining the sound produced by two isolated feathers as they were slid past each other. We performed a series of experiments to test whether (1) the velvet is responsible for reducing noise produced as a result of frictional feather rubbing interactions, and (2) the sound pressure level of rubbing noises is correlated with the amount (e.g. length) of velvet on flight feathers from a phylogenetically diverse array of bird species, from ducks to passerines. Then, for prediction 3, we compared these *in vitro* results with *in vivo* data, by comparing the sounds we recorded with the acoustic qualities of sound that birds produce when they flap their wings in flight.

## MATERIALS AND METHODS

Primary wing feathers (remiges) from 17 bird species were obtained from the Ripley Waterfowl Conservancy (https://www.ripleyconservancy.org/), the US Forest Service Barred Owl (*Strix varia*) Removal Experiment (Wiens et al., 2020), California Academy of Sciences, the San Diego Zoo, Sunshine Haven Rehabilitation (Riverside, CA, USA), March Air Force Base, from a skin in the UC Riverside teaching collection, or salvaged under California Department of Fish and Wildlife permit SC 006598 and US Fish and Wildlife Service permit MB-087454. Only feathers that did not show any signs of damage or major wear were selected for testing (listed in Table S1). For some feathers, we did not know their position in the wing. These were identified to individual position using USFWS Feather Atlas (http://www.fws.gov/lab/featheratlas/, accessed May 2019).

### Velvet anatomy

**Fig. 1.**
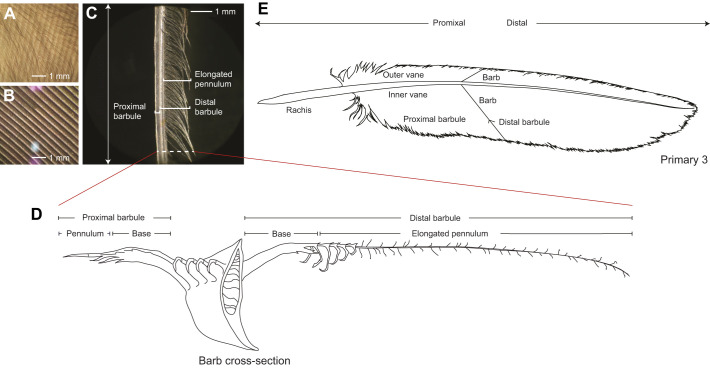
**Owl feather and velvet anatomy.** (A) Velvet on the surface of barred owl (*Strix varia*) feather p4 (location: inner vane, halfway down the length of the vane), ×30 magnification. (B) Vane of a feather that lacks the velvet (Hawaiian goose, *Branta sandvicensis*, p4, ×30 magnification). (C) Anatomical features of a single barb from a barred owl feather, showing a distal barbule with elongated pennulum, ×30 magnification. (D) Cross-section of a barb, showing the distal barbule, proximal barbule, hooklets (which attach the distal barbule to the neighboring proximal barbule), and elongated pennulum that makes up the dorsal velvet of owl feathers. Drawn by L.G.L. following Lucas and Stettenheim (1972). (E) Individual flight feathers are composed of an intricate branching system stemming from the rachis – the central shaft that barbs are attached to; they are further joined by smaller barbules projecting off it. Each barb has rows of barbules attached: the proximal barbules (bow radiates) all point towards the base of the feather while the distal barbules (hook radiates), often composed of multiple hooklets, point towards the tip of the feather. Each barbule has two segments: a base portion and a pennulum portion. The rachis divides the whole feather into the inner vane and outer vane.

We examined and photographed the proximal and distal barbules under a Leica S4 E Stereo scope at ×30 magnification, capturing feather details from the barred owl (velvet present) and Hawaiian goose (*Branta sandvicensis*) (velvet absent) (Fig. 1). Then, we quantified the length of the velvet from 24 locations in isolated feathers on different parts of the wing of barred owls (6 wings from *N*=3 birds) obtained from whole dead owls. On each of four feathers representing different parts of the wing (p10, p8, s3 and s8), 6 individual barbs were measured at positions one-quarter, a half and three-quarters of the total length of the vane, measured along the rachis by dividing the total length of the vane into quarters. At each position, two barbs, one on the outer vane and one on the inner vane, were peeled away from their neighboring barbs, flattened onto a stage plate, then photographed using an iPhone 6 mounted to the eyepiece of a Leica S4 E Stereo scope at ×30 magnification, with a ruler for scale. We then measured pennulum length in Adobe Photoshop, accounting for curvature of each barb.

For each of the 24 barb locations (4 feathers×3 vane positions×2 sides of the vane), we scored whether this location of the wing was ‘aerodynamic’ or ‘structural’. Aerodynamic regions were those that would be exposed to the boundary layer of air on the surface of a fully spread wing. Structural regions were those that were touching the feather above it, i.e. regions where the dorsal surface of the feather instead underlay an adjacent feather, and therefore was not exposed to the boundary layer of air on the surface of a spread wing. To test whether the pennulums were longer in aerodynamic or structural parts of the wing, we ran a one-way ANCOVA, with pennulum length as the dependent variable, and feather type (p10, p8, s3, s8), aerodynamic/structural and proximal vanes/distal vanes as covariates.

### Velvet presence

After conducting the in-depth assessment of velvet described above, we then investigated the presence of velvet on the remiges of all of the feathers in our possession, including the feathers of the 17 species listed in Table S1, and also common nighthawk (*Chordeiles minor*), mourning dove (*Zenaida macroura*), domestic chicken (*Gallus gallus domesticus*), Canada goose (*Branta canadensis*), argus pheasant (*Argusianus argus*), white-tailed kite (*Elanus leucurus*) and western gull (*Larus occidentalis*), for a total of 24 species. We assigned each species with presence or absence of velvet as follows.

Presence and absence were scored qualitatively (present/absent) by both sight and touch. Velvet was typically easy to spot on fully exposed feather vanes, with the longest strands of velvet reaching 3 mm in length. The dorsal surface of the feather was also felt by hand. A species was scored as having velvet if at least one flight feather had it. For instance, the American kestrel (*Falco sparverius*) exhibited a patchy distribution of velvet (velvet was not present on the outer vanes of its flight feathers) but was scored as velvet present because of the presence of velvet on the inner vanes of some of the wing feathers we examined. These were also the feathers used in the rubbing experiment, described next.

### Feather rubbing experiment

We sorted the feathers into 96 pairs of adjacent feathers (Table S1). In instances in which we obtained multiple feathers from the same bird (e.g. we plucked them from a wing ourselves), we used adjacent pairs of feathers for the rubbing experiment. However, many of our feathers were donated by sources after the feathers had molted naturally. In these instances, pairs of feathers were not necessarily from the same individual bird, but they were size and shape matched to simulate adjacent feathers within the wing, and intended to simulate how they would rub as the wings are flapped. Each feather pair formed one experimental set unit that was rubbed together for each trial.

Some of the feathers we obtained had extensive velvet (e.g. from owls), while other species had none. In addition to this natural variation, to test the effect of the velvet on rubbing sounds, we experimentally manipulated the velvet. Three experimental treatments were measured: (1) no treatment (control), (2) hairspray treatment: application of hairspray (Bed Head^®^, polymer based) on feather surfaces, which disables the hypothesized lubricating effect of the velvet by stiffening it; and (3) hairspray removed: removal of hairspray with soap and water (second control). Prior to every experimental treatment, any unhooked barbs were preened by gently pinching and stroking the misaligned barbs with neighboring ones. In the first treatment, feathers were rubbed together and measured without any further manipulation aside from simple preening. The aerosol hairspray was applied to the dorsal and ventral surfaces of the feather until all surfaces were visibly coated and color change darkened as the result of the liquid hairspray covering the surfaces and spaces in between barbs. Feathers with hairspray were allowed to set overnight. In the third experimental treatment, the feathers were run under tap water and hand soap in order to remove the hairspray. After removal of the hairspray, some feathers frayed strongly. Many barb hooking components lost the structural integrity that normally allows them to hook together with ease, and this effect seemed exacerbated in feathers with velvet. We hypothesize this was an effect of washing the hairspray off the feathers, which may have removed the preen oil from the feathers. Therefore, the third (hairspray removed) treatment did not exactly replicate the experimental conditions of the first (no manipulation) experimental treatment.

### Experimental setup

The sounds of feathers rubbing together were recorded with a Brüel & Kjær 4190 microphone (Naerum, Denmark; sensitivity: ∼50 mV Pa^−1^) attached to a Sound Devices 702 recorder (Reedsburg, WI, USA) recording at 24 bits, gain 40.0 dB, sampling at 96 kHz. The microphone was calibrated with a 94.0 dB sound pressure level (SPL) tone (1.0 kHz) played with a Brüel & Kjær sound calibrator 4231. The microphone was positioned 10 cm from the midpoint of the overlap between two adjacent feathers. The first set of experiments taken with rubbing speed as a measured variable (see below) were conducted in a normal laboratory, while the remaining experiments were performed in a sound isolation booth (Gretchken Inc.) that, although not completely anechoic, had lower levels of background sound at frequencies above 500 Hz.

Spectrograms of the rubbing sounds were analyzed in Raven Pro 1.5 (Charif et al., 2010) using the ‘average power density’ function in Raven. This function yields a time×frequency-average measure of root mean square (RMS) amplitude from within the bandwidth selected, not actual acoustic power. We analyzed recordings in the bandwidth between 1.0 to 25.0 kHz; 1 kHz was selected as a minimum frequency because most background sound fell below this frequency. We measured rubbing sounds over durations between 0.02 and 0.05 s, from the middle of each recording. This short time interval was used because it became clear that the beginning and end of each rubbing sound included transient conditions that we wanted to avoid analyzing. In addition, background sound levels were sometimes within 20 dB of the feather rubbing sound and warranted removal. Therefore, we took an estimate of the background noise level (between 1.0 and 20.0 kHz) from the recording just before the rubbing event, converted from dB to Pa using the formula level_Pa_=10^(SPL_dB_/20)^×0.00002, and subtracted the RMS background sound level from the rubbing sound level. We then converted from Pa back to dB. Finally, we calibrated the values by using the same average power density measure in Raven to digitize the 94 dB SPL calibration sound mentioned above. Thus, our measurements are presented as SPL (ref. 20 μPa) at a reference distance of 10 cm.

### Sound recordings of feather rubbing

We recorded 3 trials per treatment. From these feather rubbing sound recordings, we measured the SPL, as described above. This process was repeated for every treatment (no treatment, hairspray, hairspray removed) across feather pairs from 17 species of birds, 1–8 pairs of feathers per species, for a total of 96 pairs of feathers.

**Fig. 2.**
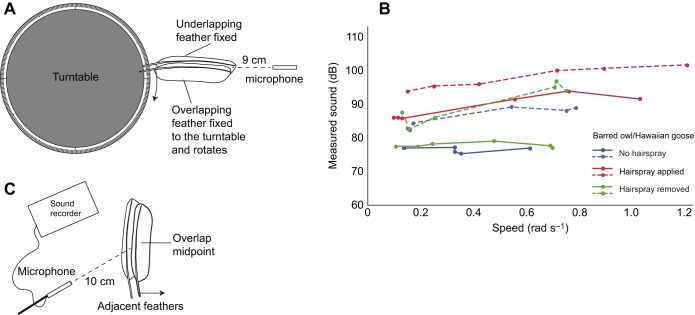
**Feather rubbing experimental design and preliminary results.** (A) The overlapping feather was affixed to a turntable and rotated with the turntable so that it slid against the underlapping feather, with a microphone 9 cm away from the midpoint of the overlapping feather region. (B) Speed of rotation of the turntable had a relatively small effect on loudness of rubbed barred owl (solid lines) and Hawaiian goose (dashed lines) feathers, with a least squares linear regression model effect size of 5.75 rad s^−1^ dB^−1^ (*n*=2 species, *n*=2 feather pairs, 6 measurements per pair per treatment). (C) In the second experimental set up, feathers were held by hand, with the overlapping region midpoint positioned 10 cm away from the microphone. While the underlapping feather remained motionless, the overlapping feather was pulled away from the underlapping feather (starting from the overlapped position) in the horizontal plane.

In preliminary iterations of the feather rubbing experiment, we sought to determine which variables affected the sound produced by two rubbing feathers as they were slid apart (Fig. 2A). The variables we considered were (1) rubbing speed, (2) the size of the feathers, which affects the surface area in contact between the two feathers as they are rubbed, and (3) normal force (the force holding the two feathers together). To test the effect of speed, we affixed the overlapping feather to the surface of a turntable that rotated radially (Fig. 2A) relative to a stationary underlapping feather. This orientation caused the feathers to slide transversely until no longer in contact. This setup allowed us to measure angular speed with video. We rotated feathers at six different speeds and three separate treatments (control, hairspray applied and hairspray removed) for one set of feathers from each of barred owl (velvet present) and Hawaiian goose (velvet absent), for a total of 36 recordings. (The goose's feathers are about the same size as barred owl feathers.) We analyzed the effect of speed with an ANOVA with bird species, experimental treatment (no hairspray, hairspray and hairspray removed) and speed as main effects, and a bird×treatment and treatment×speed interaction effect. This experiment demonstrated that the amplitude of rubbing sounds was affected by rubbing speed (see Results), but we also found that we could remove this effect by controlling speed, and controlling for speed was easy to do using the sound recordings. Specifically, for feathers of a given width, the duration of the rubbing sound was inversely proportional to the speed of rubbing; therefore, we could verify that we had held speed constant across different experimental treatments by ensuring that each individual sound of rubbing was of similar duration, for a given feather pair. Thus, in all subsequent experiments, we rubbed feathers at a constant speed, but we did not precisely measure what that rubbing speed was. Moreover, as feather width was also proportional to the duration of rubbing, measurements within a feather pair were comparable, but measurements between large (e.g. swan, barred owl) and small (e.g. poorwill, parrot) feather pairs were of intrinsically different durations, given that we had standardized rubbing speed. So, as small feathers had shorter rubbing durations, to control for feather size we only analyzed a short segment of rubbing sound from the middle of each recording (as described above).

With feathers mounted on the turntable (Fig. 2A), it was difficult to control (qualitatively) the normal force between the feathers, and it was difficult to align the surface area of the adjacent feathers so that there was uniform contact between the surfaces, as most feathers are not perfectly flat. Therefore, in subsequent experiments, the feathers were held by hand and manually rubbed together. This allowed us to rub all feather samples at the same speed while applying the same normal force while simultaneously aligning the two surfaces of different feather pairs in a way that maximized contact between surface areas, similar to how they overlap in a live bird.

To test prediction 2, as examples of frictional noise, we rubbed two pieces of sandpaper against each other as an example of frictional noise and recorded two pieces of Velcro being pulled apart as an example of interlocking. Finally, as an example of aerodynamic noise, we hand-held an Andean condor primary feather (with the broad surface of the feather perpendicular to the motion) and whipped it past a microphone inside the acoustic chamber, so that it made a turbulent whoosh sound with a good signal to noise ratio; however, this is an imperfect comparison because the distance between the feather and the microphone was not constant.

To test the effect of species, presence of velvet and the experimental manipulations, in JMP 15.0, we implemented an ANCOVA, with experimental treatment, velvet score (presence or absence) and feather identity (primary or secondary feather) as fixed effects, species ID as a random effect, a velvet×treatment interaction effect (to test the hypothesis that species that have the velvet responded to the hairspray treatment differently from species that do not) and the amplitude of sound (dB) as the dependent variable. As this analysis did not account for phylogenetic relatedness, we then did a subsequent phylogenetic analysis, using the effect sizes from this ANCOVA as inputs, as we describe next.

### Phylogenetic statistics

The dataset had both multiple samples per species as well as multiple experimental treatments per sample. As our hypothesis was that the presence of velvet interacted with the experimental effect of applying hairspray to the feathers (treatment 2) relative to the no-manipulation treatment (1) and the hairspray-removed treatment (3), we calculated the pairwise effect sizes between each of the three treatments (1 versus 3, 2 versus 1 and 2 versus 3) for each of the 17 species. As treatments 1 and 3 were both controls, we hypothesized that there would be no correlation between the effect size of 1 versus 3 against presence of velvet, while there would be a phylogenetic correlation between effect sizes of 2 versus 1 and 2 versus 3 against presence of velvet. The bird phylogeny we used was from Prum et al. (2015), pruned down to the 17 taxa for which we had experimental data.

We then exported the tree and effect size dataset to R version 4.1.2. The tree is analyzed with a phylogenetic GLS with the *ape*, *nlme* and *geiger* libraries. We conducted phylogenetic ANOVA on the presence of velvet (independent, categorical variable) against the three pairwise effect sizes (2 versus 1), (2 versus 3) and (1 versus 3), including an estimate of Pagel's λ.

### Sound recordings of birds in flight

Sound recordings of a ‘typical’ bird in flight, a black-capped chickadee (*Poecile atricapillus*) in flight were obtained from Fournier et al. (2013).

## RESULTS

### Velvet length

We found extensive velvet in the owl species we measured, and we also found the velvet in caprimulgids, hawks (e.g. red-tailed hawk, white-tailed kite) and American kestrel (Table S2). Our measurements of the velvet on three barred owl wings (Table S3) revealed the pennulums were longer within the ‘structural’ regions of the bird wings: barbs within the structural zone had an average length of 1.76±0.051 mm while barbs in the aerodynamic zone had an average length of 1.17±0.029 mm, a statistically significant difference (*F*_1140_=102.5653, *P*<0.0001). Position on the vane was also significant (*F*_2141_=27.6527, *P*<0.0001) as was vane side (outer versus inner) (*F*_1141_=31.9695, *P*<0.0001).

### Feather rubbing sounds

In the initial tests of the effect of rubbing speed, there was a statistically significant effect of speed (*F*=38.6, *P*<0.0001), treatment (*F*=83.6, *P*<0.0001), bird (barred owl versus Hawaiian goose) (*F*=166.8, *P*<0.0001) and bird×treatment (*F*=3.5, *P*=0.044) but not treatment×speed (*F*=0.45, *P*=0.63). The parameter estimate for speed was 11.5 dB change across the range of speeds we tested (Fig. 2B), from 0.2 to 1.2 rad s^−1^.

**Fig. 3.**
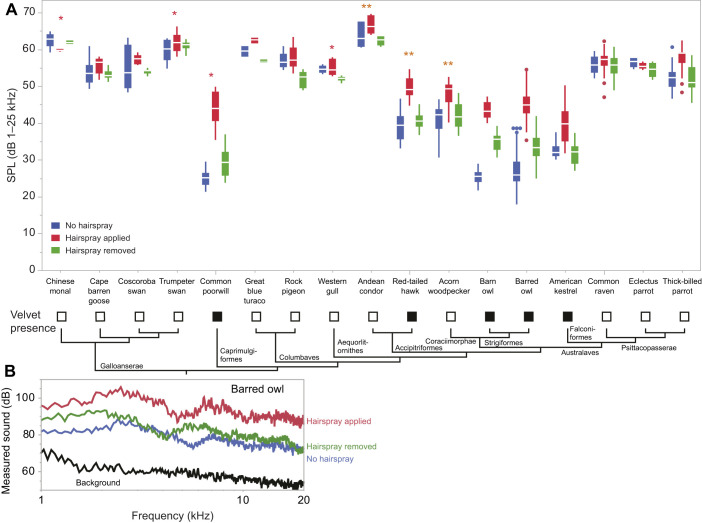
**Effects of adding hairspray on rubbing sounds of 17 species of bird.** (A) Amplitude (SPL, sound pressure level) of rubbing sounds across three experimental treatments: no hairspray (treatment 1; blue), hairspray applied (treatment 2; red) and hairspray removed (treatment 3; green). The binary presence (black squares) or absence (white squares) of velvet on flight feathers is indicated below. Box plots show 25th, median and 75th percentiles; whiskers are 1.5 times the interquartile range. See Table 2 for statistics. Phylogeny pruned from Prum et al. (2015). (B) Spectra from barred owl P5 rubbed against P6. Measured from 0.15 s of sound. Note: *y*-axis not adjusted for microphone gain settings.

Data for the full rubbing experiment on 96 pairs of feathers (*N*=17 species) rubbed by hand at constant speed are presented in Fig. 3 and Dataset 1. Five of these species had velvet, representing four convergent evolutionary origins of the velvet (two owls, one nightbird, one hawk and one falcon, American kestrel). For the full rubbing experiment, among the feather pairs measured, the barred owl primary feathers were the quietest at 18.0 dB SPL, while condor feathers were the loudest at 69.7 dB SPL (for 1–25 kHz). The full ANOVA is given in Table 2. The variable with the largest effect size was the presence of velvet: species with velvet present were on average 20.9 dB quieter than species that naturally had no velvet. Moreover, there was a significant interaction effect between the velvet presence and experimental treatment: adding hairspray increased the SPL of sounds in feathers that had velvet by 7.6 dB. Species identity (random effect) was statistically significant, and included idiosyncratic species-specific effects, including differences in feather size (surface area in contact), and additional interspecific variation in feather rubbing sound amplitude (such as Andean condor feathers, which were substantially louder than any other feathers).

**Table 2.** ANOVA model parameter estimates for the amplitude of sound of rubbing feather pairs together

Term	Sound (dB)	*t*	*P*-value
Intercept	53.8±1.35	71.2	<0.0001*
Control treatment	−0.16±0.33	−0.49	0.62
Hairspray treatment	1.74±0.33	5.2	<0.0001*
Control×velvet interaction	−7.36±0.42	−17.6	<0.0001*
Hairspray×velvet interaction	7.56±0.42	18.1	<0.0001*
Feather type (primary)	1.98±0.32	6.3	<0.0001*
Velvet (present)	−20.89±2.22	−9.4	<0.0001*

There were three experimental treatments: (1) control, (2) hairspray applied and (3) hairspray removed. Model parameters are presented relative to the third treatment (hairspray removed). Two feather types were used – primary versus secondary – and velvet was present or absent. Species identity was also included in the model as a random effect and was statistically significant (not shown). Data (means±s.e.m.) correspond to those shown by individual species/treatment plotted in Fig. 3. Asterisks indicate significant *P*-values.

As the full ANOVA did not explicitly account for phylogeny, we conducted *post hoc* phylogenetic ANOVA to test whether we got the same result when explicitly controlling for phylogeny. The effect size of applying hairspray to the feather (treatment 2) against the first control (treatment 1) was substantially greater in species with velvet present versus absent (*P*=0.0001, Pagel's λ=0.92, d.f. residual=15), as was also the case with effect size of hairspray added (treatment 2) versus removed (treatment 3) and the presence of velvet (*P*=0.0004, Pagel's λ=0.62, d.f. residual=15), as we hypothesized. Against our hypothesis, the effect size of the two controls treatment 1 against treatment 3 was also significantly correlated with the presence of velvet (*P*=0.0048, Pagel's λ=0.79, d.f. residual=15).

### Rubbing sound qualities

**Fig. 4.**
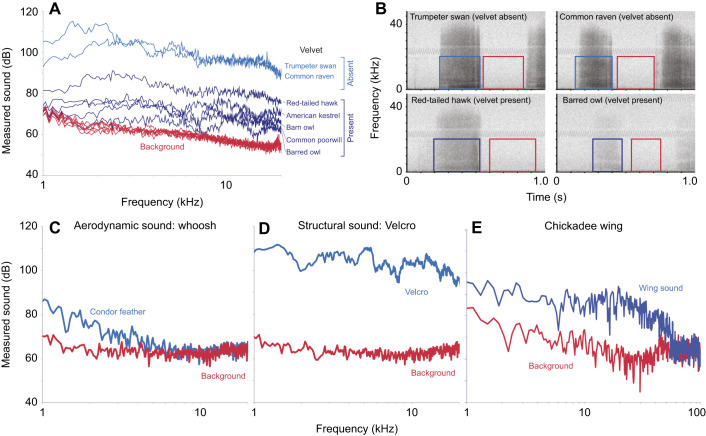
**Power spectra of sound recordings of feathers rubbing together, whooshing, Velcro being pulled apart and wing flapping of a chickadee.** (A) Power spectra (kHz, log scale) of rubbing sounds from seven species of bird (out of 17 measured in this experiment). Five species naturally have velvet (dark blue) while two species lack velvet (light blue). *N*=7 species measured, *N*=1 feather pairs per species. Note: *y*-axis not adjusted for microphone gain settings. (B) Spectrograms of rubbing trials. Blue and red boxes correspond to power spectra of rubbing (blue) and background sound (red) as shown in A. See Fig. 2C for experimental setup. Note that the microphone (Brüel & Kjær 4190) had flat sensitivity between 1 and 20 kHz, and rolled off above 20 kHz. (C) Aerodynamic whooshing sound produced by a condor primary feather swung rapidly past the microphone, where most aerodynamic sound is below 6 kHz, *N*=1 feather. (D) Sound of two strips of Velcro being pulled apart, so the hooks and loops detach from one another. Velcro produces broadband sound with approximately equal sound energy at all frequencies (white noise) between 1 and 20 kHz. *N*=1. (E) Black-capped chickadee flapping sounds (supplemental data from Fournier et al., 2013), recorded with an ultrasound (200 kHz) microphone. Wing flapping sounds include substantial energy up to approximately 70 kHz, *N*=1. For C–E, frequency is shown on a log scale.

All feathers, regardless of whether velvet was present, produced broadband sound with a relatively flat power spectrum (i.e. similar sound levels at low and high frequencies). Four examples of broadband sound production by feather rubbing are shown in Fig. 4, including power spectra (Fig. 4A) and spectrograms (Fig. 4B). The presence of velvet affected the overall sound levels but not the relative frequency distribution of SPL. For comparison, a whoosh (aerodynamic sound) produced by a condor feather whipped past a microphone is shown in Fig. 4C, and showed a declining spectrum (greatest acoustic energy at low frequency), with little acoustic energy above 6 kHz, in line with theoretical expectations for the turbulent energy spectrum (Jaworski and Peake, 2020; Rao et al., 2017). The sound of two Velcro pieces being pulled apart is shown in Fig. 4D (sandpaper, not shown, produced a similar flat spectrum). The sound of feathers rubbing (Fig. 4A) is more similar to the sound of Velcro (Fig. 4D) than to whooshing sounds (Fig. 4C). Moreover, Fig. 4E shows that the acoustic power spectrum of chickadee flight sounds from Fournier et al. (2013) remains relatively level up from 1 kHz until around 35 kHz, when the energy levels begin to decline, although there is substantial sound up to about 70 kHz.

## DISCUSSION

**Fig. 5.**
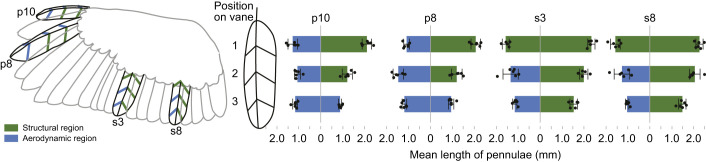
**Velvet measurements on feathers of the barred owl (*Strix varia*).** Four feathers, p10, p8, s3 and s8, from *N*=3 barred owls (both wings, i.e. *N*=6 feathers), had 6 individual distal barbules measured at three positions per feather. Blue: barbules exposed to the boundary layer on the wing, green: barbules expected to be covered by and rub against an overlapping feather (means±s.d.). Raw data are in Table S3.

Owls have a velvet coating on their feathers that contributes to their quiet flight (Fig. 1). We have presented a series of experiments that all support the SNH, that one primary function of the velvet is to reduce rubbing sounds. One line of evidence is anatomical: we found that the velvet was best developed (i.e. longer) in ‘structural’ regions characterized by overlapping feathers than the velvet in aerodynamic regions, consistent with prediction 1 (Fig. 5). American kestrels and white-tailed kites had almost no discernible velvet in aerodynamic regions, but heavy velvet in structural regions where feathers overlap (Clark et al., 2020b; Negro et al., 2006; Carlisle, 1925).

Velvet distribution across the wing has also been measured in a distant relative of barred owl, the barn owl (*Tyto alba*). Weger and Wagner (2017) measured the velvet only in wing regions exposed to airflow (explicitly avoiding measuring covered regions), including both covert and flight feathers. They found that the average length of pennulums was relatively invariant everywhere observed (793±166 μm) across exposed regions of the wing. This length is comparable to our measurements of velvet in exposed parts of barred owl wing (blue in Fig. 5) but substantially shorter than the velvet in the covered regions of the wing (green in Fig. 5). Bachmann et al. (2007) likewise found longer (>1000 μm) velvet in the proximal (covered) regions of barn owl wing feathers (see their fig. 9), and shorter velvet in exposed wing regions (see their fig. 8). Clark et al. (2020b) qualitatively measured the velvet in a handful of positions across the wing of a sample of 50 bird species, including barn owl, and found much longer velvet in covered parts of barn owl wings. In total, these data suggest that barn owls have a similar distribution of velvet across the wing to barred owls that we report here (Fig. 5). Weger and Wagner (2017) only considered aerodynamic hypotheses for the function of the velvet, and their data did not support their hypothesis that velvet length would be longer in wing regions that they hypothesized experience flow separation. We suggest that this is because the current totality of empirical evidence better supports the hypothesis that the velvet suppresses rubbing sounds (LePiane and Clark, 2020).

Our second prediction was that aerodynamic sound would tend to be greatest at lower frequencies. Our experimental sounds recapitulated these predictions: swinging a condor feather past a microphone produced a whoosh sound in which sound levels declined within increasing frequencies (the relationship between frequency and amplitude has a negative slope, Fig. 4C), while rubbing sandpaper or pulling apart two pieces of Velcro produced sound that had approximately equal sound levels across all frequencies considered (Fig. 4D), up to the roll-off frequency of our microphone (20 kHz). The sound of feathers rubbing together was similar to the sound of Velcro or sandpaper (compare Fig. 4A with Fig. 4C,D). Furthermore, the presence of velvet affected the overall sound levels but did not affect the frequency distribution of sound within the spectrum (Fig. 4A).

Our third prediction, that experimentally rubbing feathers should produce broadband sound that resembles typical flight sounds, was also supported. Notably, broadband sound that sounded similar (apart from SPL differences) was produced in all species of bird feathers that we rubbed together in our experiments (Fig. 4A). Moreover, a recording of ‘ordinary’ bird flight of a chickadee flying right next to an ultrasonic microphone (Fournier et al., 2013) revealed broadband sound up to 70 kHz. The spectrum of this chickadee wing sound was relatively flat, resembling that of the rubbing sounds we recorded. Thorpe and Griffin (1962) reported that small birds produce ultrasound in flight, whereas owls do not, which implies a relatively flat spectrum for small birds. Similarly, Boonman et al. (2020) documented white noise-like sound (what they called ‘feather noise’) in flapping flight of three passerine species and rock doves, but not three bat species. All of these observations are consistent with our hypothesis that feathers rubbing against feathers produce broadband sound that resembles white noise in flight.

Our fourth prediction was that feathers with velvet intact would produce quieter rubbing sounds than feathers without velvet. This prediction was supported: species with velvet (owls, nightbirds, hawks, falcons) produced much lower levels of sound (about 20 dB lower) than species without velvet (Fig. 3, Table 2). When hairspray was applied to the feathers, species with velvet experienced a substantial SPL increase (by about 7 dB), and when the hairspray was removed, sound levels subsided to nearly the same level as for the unmanipulated feathers. By contrast, when feathers lacking the velvet were treated with hair spray, the difference in sound level was about 1.7 dB (Fig. 3, Table 2). Our *post hoc* phylogenetic statistics returned comparable results, indicating that our results from our full statistical model were not confounded by phylogeny. These experimental results suggest that the velvet itself reduces the sound of feathers rubbing together, although as treating the velvet with hair spray produced a smaller effect (∼7 dB) than the 20 dB difference between species with and without velvet, our results also imply that species with velvet may have other unmeasured attributes that also contribute to reducing rubbing noise, such as reduced flexural stiffness, distribution (or lack thereof) of asperities (Matloff et al., 2020) or other aspects of feather texture that we did not measure.

### Mechanism

Physically, how does the velvet reduce sound produced by rubbing feathers? Akay (2002) reviewed the acoustics of friction. Feather rubbing seems to best resemble his description of stick-and-slip friction between ‘weak contacts’ comprising pairs of surfaces with many small, individual points of contact, which ‘produce light impulses as asperities come into contact and…produce a response at the natural frequencies of each component’ (Akay, 2002, p. 1526). Applying this to feathers that lack velvet, the asperities in contact between two flight feathers are barbs, barbules and cilia (Matloff et al., 2020). Assuming these asperities vary in their mechanical tuning (natural frequency) and come into contact at random intervals as the feathers slide past each other, the result should be atonal sound, consistent with the sounds we recorded (e.g. Fig. 4). What does the velvet do to modify this interaction? As the velvet fills the space in between the opposing feathers (Matloff et al., 2020), it likely replaces the asperities of the underlapping surface. It presumably has different structural tuning than the barbs and barbules. For instance, because the pennulums are smaller, perhaps they have a higher natural frequency, and/or perhaps they are more damped when the feathers slide against each other. Another possibility is that the velvet may move as the feathers slide in shear past each other, also modifying the interaction between opposing structures, similar to what Bachmann et al. (2012) showed for another feature that mediates how owl wing feathers slide past each other, the vane fringes. One open question is what effect the thickness of the velvet has on sound. Both within and among species, the velvet thickness varies from a single layer of pennulums overlying the surface of the feather to several pennulums deep (Clark et al., 2020b), but whether and how thicker velvet ameliorates more sound would be of interest.

It seemed to us that, apart from loudness differences, all of the feathers had similar acoustic qualities when rubbed, from chicken feathers to passerine feathers (Figs 3B and 4A). The condor feathers were the loudest feathers. This may have been due in part to their greater surface area, but it was our impression that their greater SPL level was more attributable to the stiffness of the vane, and other contributing aspects of feather texture that we did not quantify. We suggest that rubbing feathers sound similar because their basic geometry is the same even across a diverse phylogenetic range of birds, and thus we hypothesize typical flight noises from birds produce a similar auditory quality.

Although the birds we have focused on here appear to reduce frictional sounds, there is at least one species that appears to accentuate sound, potentially through a frictional mechanism: the magnificent riflebird (*Ptiloris magnificus*). Videos of male riflebirds performing courtship displays for females suggest they produce loud sounds resembling the rubbing of sandpaper with their wings as they slide their wings back and forth: a possible example of an accentuated frictional noise (Frith and Cooper, 1996; Laman and Scholes, 2012). Some birds, such as riflebirds (*Ptiloris* sp.) and sicklebills (*Epimachus* sp.), seem to emphasize the visual and auditory qualities of opening and closing their wings in courtship displays, producing loud feather rubbing noises, and could potentially express modified feather morphology that allows them to emphasize structural rubbing.

Although the work we present here supports the SNH over the ANH, these two hypotheses are not mutually exclusive: the velvet could reduce both types of sound. Thus, further work could better establish how prominent each acoustic effect is (sounds made by rubbing versus aerodynamic sounds) in determining the total acoustic signature of a flying bird. Here, we focused on experiments using hand-held, isolated bird feathers. This experimental paradigm has the benefit that isolated feathers are easy to manipulate, but the obvious drawback is that we have not fully established that our experiment replicates *in vivo* conditions. Two additional predictions that could be addressed in a future study of the SNH, which we were not able to test, are: that flapping flight should be louder than gliding flight; and that birds with velvet present will be quieter than birds with velvet absent, specifically in flapping flight (Clark et al., 2020a).

### Velvet diversity

Owls are not the only species with velvet: among the birds we sampled, we found it was also present in some hawks such as red-tailed hawk, nightbirds such as common poorwill and the American kestrel, constituting four separate evolutionary origins of this feature (Clark et al., 2020b; Negro et al., 2006; Carlisle, 1925).

What are the evolutionary pressures that lead to the evolution of velvet? In addition to owls, the other clades that have velvet are nightbirds (Caprimulgiformes), American kestrel and hawks (specifically, kites and harriers). Velvet is present among clades that share two different ecological similarities: nocturnality and hovering over concealed prey. Owls share nocturnality with Caprimulgiformes and kites (*Elanus* spp.), (Keirnan et al., 2022) and acoustic hunting with Circinae harriers (Rice, 1982). We have documented that species not previously widely recognized to have velvet, such as the red-tailed hawk and American kestrel, have this trait (Carlisle, 1925). Precisely why these species have it, as they are not known to be acoustic hunters, remains unclear.

## Supplementary Material

10.1242/jexbio.246234_sup1 Supplementary information

Dataset 1. Raw data
