# Supplementary material for: Acoustics of rubbing feathers: the velvet of owl feathers reduces frictional noise
Source: J Exp Biol. 2025 Jan 27;228(2):jeb246234. doi: 10.1242/jeb.246234 (PMC11832125; doi:10.1242/jeb.246234)
Supplement: Supplementary information [file jexbio-228-246234-s1.pdf]

Table S1. Feathers used in the feather rubbing experiment. The feather identities column describes specific pairs of feathers that were used in this experiment, with p denoting primary feather and s denoting secondary feather. Individual feather identity on the wing of the bird was noted when possible. ***** Indicates position of individual feather(s) identity on the wing were estimated.

code	Species	Feather Identities (sample size) (n)	Feather Origin
**CHMO**	Chinese Monal (*Lophophorus lhuysii)*	p*, p* (1); s*, s* (1)	Molted, unknown individual
**CBGO**	Cape Barren Goose (*Cereopsis novaehollandiae*)	p10, p9 (1); p8, p7 (1); p4, p3 (1); s*, s* (1)	Molted, unknown individual
**CCBS**	Coscoroba Swan (*Coscoroba coscoroba*)	p8, p7 (1); p7, p6 (1)	Molted, unknown individuals
**TRUS**	Trumpeter Swan (*Cygnus buccinator*)	p10, p9 (1); p8, p7 (1); p2, p1 (1)	Molted, unknown individual
**POWI**	Common Poorwill (*Phalaenoptilus nuttallii*)	p9, p8 (1); p4, p3 (1); p2, p1 (1); s2, s3 (1); s5, s6 (1); s7, s8 (1); s9, s10 (1)	Plucked from dead individual
**GRBT**	Great Blue Turaco (*Corythaeola cristata*)	p*, p* (1)	Molted, unknown individual
**ROPI**	Rock Pigeon (*Columba livia*)	p10, p9 (1); p8, p7 (1); p6, p5 (1); p4, p3; (1); p2, p1 (1); s1, s2 (1)	Plucked from dead individual
**WEGU**	Western Gull (*Larus occidentalis*)	P10, p* (1)	Molted, unknown individual
**COND**	Andean Condor (*Vultur grphus*)	p*, p* (1); s*, s* (1)	Molted, unknown individual
**RTHA**	Red-tailed Hawk (*Buteo jamaicensis*)	p9, p8 (1); p7, p6 (1); p4, p3 (1); p2, p1 (1); s1, s2 (1); s3, s4 (1); s5, s6 (1); s7, s8 (1)	Plucked from dead individual
**ACWO**	Acorn Woodpecker (*Melanerpes formicivorus*)	p10, p9 (1); p8, p7 (1); p6, p5 (1); p4, p3 (1); p2, p1 (1); s1, s2 (1); s3, s4 (1); s5, s6 (1)	Plucked from dead individual
**BNOW**	Barn Owl (*Tyto alba*)	p10, p9 (1); p8, p7 (1); p6, p5 (1); p4, p3 (1); p2, p1 (1); s1, s2 (1); s3, s4 (1); s5, s6 (1)	Plucked from dead individual
**BDOW**	Barred Owl (*Strix varia*) (n = 3)	p10, p9 (3); p8, p7 (3); p6, p5 (3); p4, p3 (3); p2, p1 (3); s1, s2 (3); s3, s4 (3); s5, s6 (3)	Plucked from 3 dead individuals

Journal of Experimental Biology • Supplementary information

**AMKE**	American Kestrel (*Falco sparverius*)	p10, p9 (1); p8, p7 (1); p6, p5 (1); p4, p3 (1); p2, p1 (1); s1, s2 (1); s3, s4 (1); s5, s6 (1)	Plucked from dead individual
**CORA**	Common Raven (*Corvus corax*)	p10, p9 (1); p8, p7 (1); p6, p5 (1); p2, p1 (1); s*, s* (1)	Molted, unknown individual
**ECPA**	Eclectus Parrot (*Eclectus roratus*)	p*, p* (2)	Plucked from dead individual
**TBPA**	Thick-billed Parrot (*Rhynchopsitta pachyrhyncha*)	p8, p7 (1); p6, p5 (1); p2 p1 (1); s1, s2 (1); s3, s4 (1); s5, s6 (1); s7, s8 (1)	Plucked from dead individual

Journal of Experimental Biology • Supplementary information

Table S2. Velvet presence across a wide taxonomical range.

Species	Remiges examined (sample size when n > 1)	Has velvet on remiges?
Chinese Monal (*Lophophorus lhuysii)*	p* (2); s* (2)	absent
Great Argus (*Argusianus argus*)	P*, s* (2)	absent
Domestic Chicken (*Gallus gallus domesticus*)	p10, p9, p8, p7, p6, p5, p4, p3, p2, p1, s1, s2, s3, s4, s5, s6	absent
Canada Goose (*Branta canadensis*)	p9, p8, p7, p5, p2	absent
Cape Barren Goose (*Cereopsis novaehollandiae*)	p10, p9, p8, p7, p4, p3, s* (3)	absent
Coscoroba Swan (*Phalaenoptilus nuttallii*)	p8, p7 (2), p6	absent
Trumpeter Swan (*Cygnus buccinator*)	p10, p9, p8, p7, p6, p3, p2, p1, s1	absent
Common Nighthawk (*Chordeiles minor*)	p10, p9, p8, p7, p6, p5, p4, p3, p2, p1, s1, s2, s3, s4, s5, s6, s7, s8	present
Common Poorwill (*Phalaenoptilus nuttallii*)	p9, p8, p6, p4, p3, p2, p1, s2, s3, s5, s6, s7, s8, s9, s10	present
Great Blue Turaco (*Corythaeola cristata*)	p* (2), s*	absent
Mourning Dove (*Zenaida macroura*)	p8, p7, p6, p5, p4, p3	absent
Rock Pigeon (*Columba livia*)	p10, p9, p8, p7, p6, p5, p4, p3, p2, p1, s1, s2, s3	absent
Western Gull (*Larus occidentalis*)	p9, p8, p7, p6	absent
Secretary bird (*Sagittarius serpentarius*)	p* (3), p* (2)	absent
Andean Condor (*Vultur gryphus*)	p* (2), s* (2)	absent
Red-tailed Hawk (*Buteo jamaicensis*)	p10, p9(2), p8 (2), p7 (2), p6 (2), p4 (2), p3 (2), p2 (2), p1 (2), s1 (2), s2 (2), s3 (2), s4 (2), s5 (2), s6 (2), s7 (2), s8 (2), s9 (2), s10 (2), s11	present
White-tailed Kite	p9, p8	present

Journal of Experimental Biology • Supplementary information

(*Elanus leucurus*)		
Acorn Woodpecker (*Melanerpes formicivorus*)	p10, p9, p8, p7, p6, p5, p4, p3, p2, p1, s1, s2, s3, s4, s5, s6, s7, s8	absent
Barn Owl (*Tyto alba*)	p10, p9, p8, p7, p6, p5, p4, p3, p2, p1, s1, s2, s3, s4, s5, s6, s7, s8, s9, s10	present
Barred Owl (*Strix varia*)	p10 (4), p9 (4), p8 (4), p7 (4), p6 (4), p5 (4), p4 (4), p3 (4), p2 (4), p1 (4), s1 (4), s2 (4), s3 (4), s4 (4), s5 (4), s6 (4), s7 (4), s8 (4), s9 (4), s10 (4)	present
American Kestrel (*Falco sparverius*)	p10, p9, p8, p7, p6, p5, p4, p3, p2, p1, s1, s2, s3, s4, s5, s6, s7, s8, s9, s10	present
Common Raven (*Corvus corax*)	p10, p9, p8, p7, p6, p5, p2, p1, s* (6)	absent
Eclectus Parrot (*Eclectus roratus*)	p* (4)	absent
Thick-billed Parrot (*Rhynchopsitta pachyrhyncha*)	p10, p8, p7, p6, p5, p4, p2 p1, s1, s2, s3, s4, s5, s6, s7, s8, s9	absent

Journal of Experimental Biology • Supplementary information

Table S3. Velvet Length across the wings of the Barred Owl (*Strix varia*).

feather	position	structural or aerodynamic region	Mean pennulum length (mm)	std dev
**p10**	1 outer	a	1.28	0.21
**p10**	1 inner	s	2.1	0.14
**p10**	2 outer	a	1.06	0.14
**p10**	2 inner	s	1.21	0.23
**p10**	3 outer	a	1.12	0.11
**p10**	3 inner	a	1.21	0.23
**p8**	1 outer	s	1.09	0.13
**p8**	1 inner	s	2.06	0.21
**p8**	2 outer	a	1.47	0.24
**p8**	2 inner	a	1.20	0.24
**p8**	3 outer	a	1.47	0.24
**p8**	3 inner	a	0.94	0.12
**s3**	1 outer	s	1.6	0.16
**s3**	1 inner	s	2.3	0.17
**s3**	2 outer	a	1.35	0.35
**s3**	2 inner	s	1.99	0.19
**s3**	3 outer	a	1.16	0.09
**s3**	3 inner	s	1.53	0.17
**s8**	1 outer	s	1.57	0.22
**s8**	1 inner	s	2.26	0.16
**s8**	2 outer	a	1.29	0.36
**s8**	2 inner	s	2.05	0.24
**s8**	3 outer	a	1.03	0.08
**s8**	3 inner	s	1.48	0.17

Dataset 1. Raw data

Available for download at
https://journals.biologists.com/jeb/article-lookup/doi/10.1242/jeb.246234#supplementary-data

Journal of Experimental Biology • Supplementary information
